# Circulating Levels of Omentin, Leptin, VEGF, and HGF and Their Clinical Relevance with PSA Marker in Prostate Cancer

**DOI:** 10.1155/2018/3852401

**Published:** 2018-08-16

**Authors:** M. Fryczkowski, R. J. Bułdak, T. Hejmo, M. Kukla, K. Żwirska-Korczala

**Affiliations:** ^1^Department of Urology, Public Health Care Provincial Specialist Hospital, No. 3, Rybnik, Poland; ^2^Department of Biochemistry, School of Medicine with the Division of Dentistry, Medical University of Silesia, Zabrze, Poland; ^3^Department of Gastroenterology and Hepatology, School of Medicine in Katowice, Medical University of Silesia, Katowice, Poland; ^4^Department of Physiology, School of Medicine with the Division of Dentistry, Medical University of Silesia, Zabrze, Poland

## Abstract

**Background:**

Prostate cancer (PCa) is the first in terms of occurrence in Europe and second in Poland. The PCa risk factors include: genetic load, obesity, diet rich in fat, hypertriglyceridemia, and exposure to androgens. The prostate-specific antigen (PSA) level may be elevated in prostate cancer or other prostate disorders. Fat tissue secretes adipocytokines, which increase the risk of cancer development and metastasis.

**Objectives:**

The aims of the study were to investigate the relationship between circulating levels of PSA, adipocytokines: omentin, leptin, hepatocyte growth factor (HGF), and vascular endothelial growth factor (VEGF) in serum obtained from patients with benign prostate hyperplasia (BPH) and prostate cancer (PCa).

**Methods:**

Forty patients diagnosed with BPH and forty diagnosed with PCa were assessed for the purpose of the study. The concentrations of omentin, leptin, HGF, and VEGF were determined using enzyme-linked immunosorbent assays (EIA).

**Results:**

PSA level was significantly higher in the PCa group than in BPH (18.2 versus 9 ng/mL, *p* < 0.01), while volume of prostate gland was significantly higher in the BPH group than in PCa (39.1 versus 31.1 cm^3^, *p* = 0.02). HGF, VEGF, omentin, and leptin concentrations were significantly higher in PCa group than in BPH (359.5 versus 294.9 pg/mL, *p* = 0.04; 179.3 versus 127.3 pg/mL, *p* < 0.01; 478.8 versus 408.3 ng/mL, *p* = 0.01; 15.7 versus 11.2 ng/mL, *p* = 0.02, resp.). The multiple logistic regression analysis demonstrated that only omentin and PSA levels were independent predictors of PCa in studied subjects.

**Conclusions:**

PSA level as well as the level of omentin may be valuable markers of PCa with clinical significance, when compared to PSA.

## 1. Introduction

Prostate cancer (PCa) is the second most common (above 13%) type of cancer in Polish male population after lung cancer. The main risk factor is age, PCa develops most frequently in patients older than 50 years of age. Prostate cancer has mostly glandular structure and is androgen-dependent. Prostate cancer is usually suspected on the basis of digital rectal examination (DRE) and/or prostate-specific antigen (PSA) level. PSA plays a crucial role in diagnosing prostate cancer [1]. The PSA concentration in serum of healthy men is between 0.1 and 4 ng/mL [2]; however, it strongly depends on the volume of prostate. Its value increases not only in prostate diseases (prostatitis and benign prostate hyperplasia), or cystitis, but also in other cancers with inflammatory states. Many reports show a low specificity of PSA [3, 4], false-positive [5, 6] or false-negative [7] results. On the other hand, Shoaibi et al. [8] found PSA to be a very specific and sensitive biomarker of high-risk PCa. Therefore, it is important to find other markers, which would facilitate more precise diagnosis and management.

The promotion of different cancers, that is, prostate, ovarian, or colorectal one is related not only with obesity but also with overweight [9, 10]. Finding adipose tissue-derived hormones (adipokines) linking obesity and prostate cancer is one of the most important topics in current research. Defining their functions, molecular targets, and potential clinical value, as in the case of PCa and its treatment biomarkers, is crucial. Adipokines have a broad spectrum of activities and may influence many processes including inflammation, various metabolic pathways, angiogenesis, cell growth, proliferation, and migration both in normal and neoplastic cells.

Omentin is a newly discovered plasma adipokine expressed by adipose tissue stromal vascular cells [11]. In contrast to other adipokines, its plasma concentration is lower in patients with obesity, insulin resistance [12], and diabetes mellitus type 1 and type 2 [13, 14]. Its level is elevated in inflammatory states, nonalcoholic fatty liver disease, cirrhosis, portal vein thrombosis, and chronic hepatitis C [13, 15, 16]. The role of omentin in cancer has not been established yet. Some researchers claim that it may suppress growth, migration, and invasion of gastric cancer cells *in vitro* and tumor growth and metastasis *in vivo* by upregulating the hepatocyte nuclear factor 4 alpha (HNF4*α*) [17], promoting apoptosis in human hepatoma cells *in vitro* [18], and acting as anti-inflammatory factor in rats [19]. The published results of *in vivo* studies are limited to the assessment of plasma concentrations of omentin in prostate [20], gastric [17, 21], colorectal [22–25], renal [26], and pancreatic cancer [27]. The high level of circulating omentin in patients with colorectal cancer seems to be a good prognostic factor [21, 23].

Leptin is a cytokine produced mostly by adipocytes and is mainly responsible for the regulation of energy balance and food intake [28]. Leptin concentration may be increased in obese and diabetic patients [29]. Leptin receptor (ObR) may activate genes responsible for cell proliferation and angiogenic factors such as VEGF [30, 31]. Leptin is expressed and synthesized by prostate and is involved in normal physiological growth of prostate gland [32]. Overexpression of leptin may result in extensive proliferation and, ultimately, prostate hyperplasia or cancer [33].

Angiogenesis is crucial for tumor growth and is controlled by many growth factors, cytokines, and hormones [34]. The vascular endothelial growth factor is essential in pathological angiogenesis, and its gene expression is upregulated by hypoxia occurring in solid tumors [35]. VEGF-secreting tumors are able to grow rapidly and metastasize. Solid tumors require consistent angiogenesis together with tumor growth to supply them with nutrients and oxygen. High concentration of VEGF in plasma of patients with tumor results in poor prognosis.

Hepatocyte growth factor (HGF) is another molecule inducing tumor angiogenesis [36]. It was initially identified as a potent hepatotrophic factor responsible for liver regeneration [37], but now its other functions like mediating tumor-stromal interaction with morpho-, moto-, and mitogenic activities have become known [38, 39]. Moreover, HGF intensifies the potential angiogenic activity in vascular endothelial cells [40]; it also acts as a paracrine factor responsible for morphogenesis, cell growth, and cell motility. It has been shown that cancer-associated fibroblasts promote cell scattering, epithelial-mesenchymal transition (EMT), and migration of cancer cells in an HGF-dependent manner. Both HGF and c-Met are upregulated in different types of human cancers such as breast, lung, colorectal, gastric, and oesophageal cancer [41–43].

### 1.1. Objectives

The main aims of the study were to assess the circulating level of leptin and omentin as well as related proteins: HGF and VEGF in men with prostate cancer and benign prostate hyperplasia, and to study the associations between their concentrations and PSA level. An additional target was to assess their utility as noninvasive biomarkers useful in diagnosing prostate cancer.

## 2. Patients and Methods

### 2.1. Patients

Forty patients diagnosed with benign prostatic hyperplasia (BPH) and 40 diagnosed with PCa after evaluation were selected for the research study. Basic physical and *per rectum* examinations have been performed and medical history has been collected. The patients from both groups were between 55 and 75 years of age. After biopsy the patients were assigned to either PCa or BPH group. The patients assigned to BPH group with high level of PSA were referred to another biopsy and excluded from final qualification if prostate cancer was diagnosed.

### 2.2. Inclusion Criteria

Patients with PSA ≥ 4 and ≤ 40 ng/dL, BMI < 30 kg/m^2^, and prostate hyperplasia or prostate cancer with Gleason score of 6-7 have been included. Informed consent was obtained from each patient.

### 2.3. Exclusion Criteria

Patients with other cancers, acute genitourinary system necrosis, previous prostate operations, presence of hemodynamic disturbances precluding performing examination, type 1 and type 2 diabetes mellitus, chronic liver and kidney diseases, mental disorders, and obesity were excluded from the research.

### 2.4. Sample Collection

All patients were treated with ciprofloxacin 500 mg in pills twice a day for 5 days to provide biopsy safety. All patients with PCa had prostate ultrasound-guided biopsy (transrectal ultrasound-guided biopsy TRUS-Bx). After fasting overnight and 48 h after antibiotic treatment 4 mL of blood was collected. There were 6 to 12 biopsy specimens collected from each patient. Biopsies were fixed in formalin for histopathological evaluation. Blood samples were centrifuged (serum/plasma) and frozen at −20°C and transported, in proper containers, to the laboratory in Physiology Department of Medical University of Silesia in Zabrze, Poland, and stored in −70°C until evaluation.

### 2.5. Serum Levels of Omentin, Leptin, HGF, and VEGF

Concentrations of biomarkers were measured using immunoenzymatic assays (ELISA) in duplicate at the same time, according to the manufacturers' protocol.

The concentration of leptin was measured using BioVendor kit with 0.2–50 ng/mL detection level and 5.6% of inter- and 5.9% of intra-assays coefficients of variations.

The level of omentin was determined with BioVendor kit with 0.5–64 ng/mL detection level and 4.4% of inter- and 3.2% of intra-assays coefficients of variations.

Concentration of HGF was measured using RayBiotech kit with minimum detectable dose 3 pg/mL and < 12% of inter- and < 10% of intra-assays coefficients of variations.

The level of VEGF was determined with Gen-Probe kit with 7.9–200 pg/mL detection level and 4.3% of inter- and 6.2% intra-assays coefficients of variations.

### 2.6. Statistical Analysis

The database of clinical material was made in Microsoft Excel v. 2010. All calculations were made in StatSoft Statistica v. 7.1PL and MedCalc Statistical Software v. 14.10.2. The distribution of variables was determined with the use of Shapiro-Wilk's test. Descriptive values are presented as means and standard deviations for normally distributed, and medians and quartiles for other than normally distributed. Student's *t*-test was used to compare the level of triglycerides (TG) and low density lipids (LDL) which were normally distributed. Other variables that were not normally distributed were compared using Mann–Whitney *U* test. Statistical significance was indicated for *p* < 0.05.

The study was approved by the Bioethical Committee of the Medical University of Silesia (approval symbol KNW/0022/KB1/182a/11).

## 3. Results

Table 1 shows the characteristics of analyzed patients. Both groups were adjusted to age and BMI.

Serum concentrations of HGF (359.5 versus 294.9 pg/mL; *p* = 0.04), VEGF (179.3 versus 123.3 pg/mL; *p* = 0.04), leptin (15.7 versus 11.2 ng/mL; *p* = 0.02), omentin (478.8 versus 408.3 ng/mL; *p* = 0.03), prostate-specific antigen (18.2 versus 9 ng/mL; *p* < 0.01) are significantly higher in PCa than in BPH group. The prostate volume was significantly higher in BPH group than in PCa (39.1 versus 31.1 cm^3^; *p* = 0.02). Other parameters were not statistically significant.

**Figure 1:**
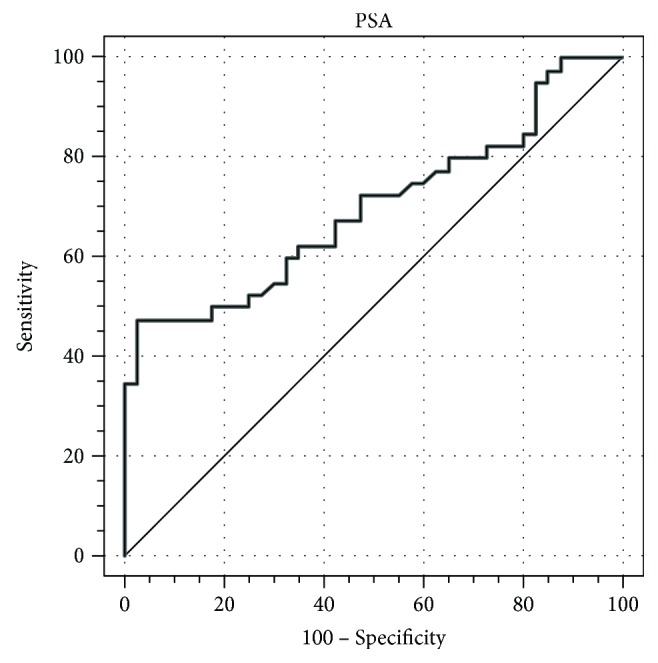
ROC curve for PSA.

**Figure 2:**
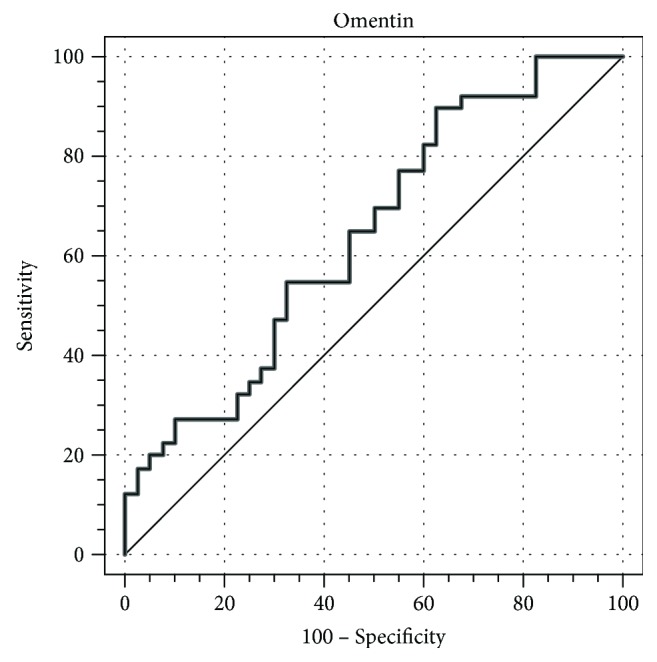
ROC curve for omentin.

Figures 1 and 2 present the cut-off limits of PSA and omentin, respectively. ROC curves illustrate the sensitivity and specificity for diagnosis of PCa.

ROC curve of PSA concentration shows a fair discriminant power with AUC = 0.70 (95% CI: 0.587–0.797) for differentiation between PCa and BPH with very high specificity and moderate sensitivity (97.5% and 47.5%, resp., cut-off value: 21.9 ng/mL).

The ROC curve for omentin shows a poor discriminant power for differentiation between PCa and BPH with AUC = 0.593 (95% CI: 0.477–0.702), high sensitivity and low specificity (90.0% and 37.5%, resp., cut-off value: 343.18 ng/mL).

According to the univariate logistic regression, the elevation of each biomarker presented in Table 2 (omentin, leptin, VEGF, HGF, and PSA) suggests a statistically significant higher risk of prostate cancer development (OR: 1.005, *p* = 0.01; OR: 1.053, *p* = 0.02; OR: 1.010, *p* < 0.01; OR: 1.003, *p* = 0.03; OR: 1.090, *p* = 0.01, resp.).

Table 3 presents correlations between analyzed biomarkers. We noted that there were strong positive correlations in PCa and BPH groups between leptin and VEGF (*R*_S_ = 0.722, *p* < 0.01; *R*_S_ = 0.802, *p* < 0.01) and VEGF and HGF (*R*_S_ = 0.845, *p* < 0.01; *R*_S_ = 0.775, *p* < 0.01). Strong positive correlations in PCa groups occurred for VEGF and PSA (*R*_S_ = 0.867, *p* = <0.01) and PSA and HGF (*R*_S_ = 0.755, *p* < 0.01). Other positive correlations were moderate.

The final step of multivariate backward stepwise logistic regression showed in Table 4 presents the weakest correlation between PSA and omentin and higher risk for prostate cancer (OR: 1.091, *p* < .01; OR: 1.005, *p* = 0.01, resp.).

## 4. Discussion

In the present study, the patients with PCa had significantly higher concentrations of circulating PSA, HGF, VEGF, omentin, and leptin as well as significantly lower volume of prostate gland than patients with BPH. We provide the first clinical evidence demonstrating that PSA levels along with omentin may be valuable markers of PCa with clinical significance, when compared to PSA only. Omentin is a relatively novel adipocyte-derived cytokine mainly expressed in visceral adipose tissues. The serum omentin level is decreased in patients with obesity, insulin resistance, and type 1 and type 2 diabetes [12–14].

Elder people often have the HOMA-IR (overnight fasting insulin to glucose ratio) level higher than 1, which is diagnosed as insulin resistance (IR); however, it may result from simultaneously increased level of insulin and decreased level of glucose and is not equivalent with type 2 diabetes. For this reason, we selected homogenous study participants in both groups (PCa and BPH) who have similar adiposity level according to BMI level and similar HOMA-IR value. Patients in both groups had normal weight or were slightly overweight and HOMA-IR indicates insulin resistance in most of them. The dependence between HOMA-IR and associated risk of prostate cancer is still not clear. Stocks et al. claim that insulin resistance is inversely related to nonaggressive prostate cancer and nonsignificantly positively related to the risk of aggressive disease [44]. On the other hand, Yun et al. found a positive association of elevated HOMA-IR and the risk of locally advanced prostate cancer with the risk increasing with increasing IR [45]. Many reports show that there is an inverse association between type 2 diabetes mellitus (T2DM) and the risk of prostate cancer [46–49]. Interestingly, Bensimon et al. found T2DM to be associated with increased risk of prostate cancer mortality [50].

Such a selection of patients, together with type 1 and type 2 diabetes as exclusion criterion, allows us to compare both groups with prostate cancer tissue as main source influencing the level of omentin.

We observed some increase in the level of omentin in PCa patients when compared to the patients with BPH (478.8 versus 408.3 ng/mL, resp.). Our study confirms the previous data published by Uyeturk et al. who showed the increase of circulating omentin level in patients with PCa compared to those BPH in Turkish population (546.8 versus 373 ng/mL) [20]. However, in Uyeturk et al.'s study there is a significant difference in the size of the BPH and PCa groups, as well as a difference in BMI, with a significantly higher value for PCa patients. Omentin level has been previously proven to be obesity-related (negative correlation) [14, 26], and it could have been be the source of increased difference in Uyeturk et al.'s data compared to our study, in which we adjusted patients according to BMI. According to the ROC curve of omentin, this biomarker shows poor discriminant power for differentiation between PCa and BPH. The increase of omentin concentration statistically significantly increases the risk of PCa (OR: 1.005, *p* = 0.01). Aleksandrova et al. reported higher level of circulating omentin in patients with colorectal cancer in a prospective cohort study [25]. An increased level of circulating omentin was reported also in colon and colorectal cancer, gastric cancer, malignant pleural mesothelioma, and pancreatic adenocarcinoma [21, 22, 24, 27, 51], while in renal cell carcinoma it has been significantly lower as compared to control [26]. It could possibly mean that higher levels of omentin in serum of PCa patients may result not only from adipose tissue but also from cancer tissue. The role of omentin in cancer progression has not been established yet, but most researchers claim it may be an anticancer factor. Unlike them, we hypothesize that cancer tissue may produce omentin to increase insulin-induced glucose intake in tumor, as omentin increases tissue sensitivity to insulin.

In our study, the patients with PCa demonstrated significantly higher levels of PSA than patients with BPH (18.2 versus 9 ng/mL, resp.). Similar results in prostate cancer patients were obtained by Saǧlam et al., while the concentration of PSA in patients with BPH and healthy control was higher than in our study (3.17 versus 3.36 ng/mL, resp.) [52]. The ROC curve for PSA shows a fair level discriminant power with AUC = 0.70 (95% CI: 0.587–0.797) for differentiation between PCa and BPH with high specificity (97.5%) and moderate sensitivity (47.5%). We found significant positive correlations between PSA and other biomarkers such as HGF and VEGF in the PCa group and positive one with leptin in both PCa and BPH groups. The increase of PSA concentration in serum generates a statistically significant increase of risk for PCa occurrence (OR = 1.090, *p* = 0.01). Hashem and Essam found a significantly higher level of circulating PSA in patients with localized and metastatic prostate cancers than in healthy controls (17.4 versus 96.0 versus 1.8 ng/mL, resp.) [53]. Mean PSA concentration levels in localized tumor and ROC curve specificity are close to those obtained in our study (17.4 versus 18.2 ng/mL; 100 versus 97.5%, resp.); however, the sensitivity was higher than in our results (100% versus 47.5%).

As we described it in the introduction, PSA is still the only widely used prognostic biomarker of prostate cancer, despite the disadvantages reported. On the other hand, omentin concentration may be affected by many illnesses or the physiology of patient. Thanks to restrictive selection of patients, we were able to compare omentin concentration with the assumption that its level is influenced mostly by cancer tissue. Notably, our multiple logistic regression analysis demonstrated that only omentin and PSA levels were independent predictors of PCa in the studied subjects.

Leptin concentration levels turned out to be significantly higher in patients with PCa compared to patients with BPH (15.7 versus 11.2 ng/mL, resp.). As has been mentioned above, both groups have had similar BMI levels. Some studies show no significantly higher concentration of serum leptin in prostate cancer, compared to healthy patients or patients with benign prostate hyperplasia [54], or in prostate tumor patients [55], while others show its concentration to be higher [52, 56]. The association of leptin with the risk of PCa occurrence or its stage is also disputable. There are some studies which did not reveal any relationship in that respect [57–59], while one found the relationship to be positive [60]. Grosman et al. assessed the serum level of leptin in healthy men and in patients with PCa and BPH [61]. The patients were divided into groups, with and without a metabolic syndrome. The total PSA level in the BPH group was significantly higher than in control group and significantly lower than in PCa group. The median level of leptin in patients without metabolic syndrome was higher in the PCa group than in BPH group, but the difference was not statistically significant.

Also in our study, the patients with PCa revealed a significantly higher concentration of leptin than the patients with BPH. Those results are consistent with those achieved by Singh et al. [56]. However, Singh et al. found no correlation between leptin and PSA, while in our study such a relationship was positive in both PCa and BPH groups. Also, results obtained by Saǧlam et al. match with our results (27.33 versus 16.96 versus 17.55 ng/mL, PCa versus BPH versus healthy control, resp.) [52]. Strong positive correlation between PSA and leptin has been presented in Saǧlam et al.'s study, and it is similar to our data. Serretta et al. found that circulating leptin has the highest diagnostic accuracy with AUC = 0.781 of ROC curve [62]. The circulating leptin concentration is higher in patients with breast [63], gastric [30], and endometrial [64] cancer, but it is lower in pancreatic cancer [65]. Meta-analysis revealed a higher concentration of serum leptin in patients with endometrial cancer than in healthy controls [66], and the association of decreased leptin concentration with reduced risk of endometrial cancer [64].

In our study, the patients with PCa demonstrated a significantly higher concentration of serum VEGF (179.3 versus 123.3 pg/mL, resp.). The results obtained by Skerenova et al. had shown higher levels to have occurred in men with castration resistant prostate cancer (CRPC) than in healthy men (332 versus 239 pg/mL); however, the median concentration in PCa group was much higher than in our results (332 versus 179.3 pg/mL) [67]. It may result from the assortment of patients, as Skerenova et al. examined patients with highly advanced cancer. The meta-analysis by Kut et al. gathered results from 13 studies measuring serum and plasma level of VEGF in patients with prostate cancer [68]. The weighted average in healthy patients' serum was 129 pg/mL and 281 pg/mL for PCa. The concentration of VEGF in the BPH group in our study is consistent with this analysis, while in PCa group it is lower. VEGF inhibitors are known to suppress prostate cancer cell migration *in vivo* [69]. VEGF is also produced by adipose tissue, where it is involved in vascularization [70]. However, Yin et al. found that HGF was produced by adipocytes and macrophages, while VEGF was produced mainly by mature adipocytes [71]. The circulating HGF was assessed as a potential prostate cancer biomarker by Yasuda et al. [72].

In our study, the concentration of HGF in serum was significantly higher in PCa patients than in those with BPH (359.0 versus 294.9 pg/mL, resp.). Hepatocyte growth factor (HGF) is an important, multipurpose factor, which may promote proinflammatory conditions and tumorigenesis. Nishida et al. studied the expression of HGF by prostate cancer tissues and linked higher concentration of HGF with higher risk of cancer recurrence [73]. Recent studies also show that HGF is produced by adipose tissue, which results in higher concentration of HGF in serum of obese patients [74]. Yasuda et al. measured the concentration of active HGF (AHGF) in serum of patients with different grading of prostate cancer and benign prostate hyperplasia, and they found the concentration of HGF to be significantly higher in the cancer group (0.36 versus 0.28 ng/mL, resp.) and to be increasing together with the tumor grading [72]. Moreover, Nagakawa et al. also found a significantly higher concentration of HGF in serum of patients with untreated prostate cancer than in those with benign prostate hyperplasia (1.56 versus 1.07 ng/mL, resp.) [75]. The concentrations of serum HGF measured by Nagakawa et al. turned out to be higher than in our study, but the relationship remains similar. The study by Yin et al. shows increased expression of *c-Met*, the HGF receptor in prostate cancer cells *in vitro,* in metastatic-dependent manner [76]. It allows to use HGF as a potential indirect prostate cancer biomarker. The concentration of HGF may not only be a PCa presence marker but also a marker of its advance. Dayyani et al. showed that a higher HGF concentration in serum was associated with a shorter progression-free survival (PFS) [77].

Taken together, as a cancer marker, omentin—along with PSA level—turned out to be helpful in diagnosing prostate cancer, especially in nonobese patients with insulin resistance.

Our study has a few limitations. Additional studies are required to determine whether the levels of omentin are increased in PCa patients as a compensatory mechanism against increased IR or as a manifestation of poor omentin metabolism in the cancer tissue. Omentin expression in PCa and BPH and its potential role in cancer pathology also requires clarification. We should also keep in mind that our prostate cancer patients had the Gleason score of 6-7; therefore, we could not provide data for patients with Gleason score <6 or >7.

## Figures and Tables

**Table 1:** Clinical characteristics in prostate cancer (PCa) and benign prostate hyperplasia (BPH).

Parameter	PCa *n* = 40	BPH *n* = 40	*p* value
Age (yrs)	70.5 (64–73)	67.4 (62.5–68.5)	0.51^a^
BMI (kg/m^2^)	26.4 (23.7–28.0)	26.4 (25.2–29.1)	0.20^a^
T-Chol (mg/dL)	189.0 (170.0–220.0)	190.5 (161.5–199.5)	0.16^a^
HDL (mg/dL)	39.5 (31.0–55.0)	40.0 (29.5–55.0)	0.78^a^
TG (mg/dL)	152.6 ± 54.2	143.0 ± 43.6	0.38^b^
LDL (mg/dL)	121.1 ± 28.7	123.0 ± 34.2	0.69^b^
Glucose (mg/dL)	121.1 (104.0–126.5)	122.0 (108.5–132.0)	0.92^a^
Insulin (IU/dL)	8.0 (5.7–11.0)	9.8 (6.8–15.4)	0.06^a^
HOMA-IR	2.3 (1.7–3.7)	2.8 (1.8–4.9)	0.12^a^
Omentin (ng/mL)	478.8 (398.2–584.7)	408.3 (323.3–528.0)	0.03^b^
Leptin (ng/mL)	15.7 (8.2–26.8)	11.2 (6.0–16.0)	0.02^b^
VEGF (pg/mL)	179.3 (116.0–266.0)	123.3 (70.8–192.7)	<0.01^b^
HGF (pg/mL)	359.5 (266.6–462.9)	294.9 (192.1–353.7)	0.04^b^
PSA (ng/mL)	18.2 (7.7–30.8)	9 (5.5–17)	<0.01^a^
Prostate volume in TRUS (cm^3^)	31.1 (20.0–38.2)	39.1 (24.9–47.5)	0.02^a^

Significant differences of examined parameters between groups have been bolded. Statistic tests: ^a^Mann–Whitney's *U* test, ^b^Student's *t*-test. PCa: prostate cancer; BPH: benign prostate hyperplasia; BMI: body mass index; T-Chol: total cholesterol; HDL: high-density lipoprotein; TG: triglycerides; LDL: low-density lipoprotein; HGF: hepatocyte growth factor; VEGF: vascular endothelial growth factor.

**Table 2:** Univariate logistic regression of selected biomarkers including odds ratio.

Dependent variable: PCa; independent variable: omentin					
Level of statistical significance of the model: *p* < 0.01					
Variable	Coefficient	Standard error	*p*	Odds ratio	95% confidence interval
Omentin	0.005	0.002	0.01	1.005 (40.7)	1.001 to 1.008
Constant	−2.124	0.864	0.01		
Dependent variable: PCa; independent variable: leptin					
Level of statistical significance of the model: *p* < 0.01					
Variable	Coefficient	Standard error	*p*	Odds ratio	95% confidence interval
Leptin	0.051	0.021	0.02	1.053 (20.97)	1.009 to 1.098
Constant	−0.820	0.340	0.04		
Dependent variable: PCa; independent variable: VEGF					
Level of statistical significance of the model: *p* < 0.01					
Variable	Coefficient	Standard error	*p*	Odds ratio	95% confidence interval
VEGF	0.008	0.003	<0.01	1.010 (112.8)	1.002 to 1.014
Constant	−1.320	0.503	<0.01		
Dependent variable: PCa; independent variable: HGF					
Level of statistical significance of the model: *p* < 0.01					
Variable	Coefficient	Standard error	*p*	Odds ratio	95% confidence interval
HGF	0.003	0.001	0.03	1.003 (52.4)	1.000 to 1.006
Constant	−0.991	0.507	0.05		
Dependent variable: PCa; independent variable: PSA					
Level of statistical significance of the model: *p* < 0.01					
Variable	Coefficient	Standard error	*p*	Odds ratio	95% confidence interval
PSA	0.082	0.025	0.01	1.090 (47.4)	1.034 to 1.140
Constant	−1.226	0.426	<0.01		

**Table 3:** Correlations between the analyzed variables.

Correlated variables	Group	Omentin	VEGF	HGF	PSA
Leptin	PCa	*R*_s_ = −0.640 *p* = 0.69	*R*_s_ = 0.722 *p* < 0.01	*R*_s_ = 0.673 *p* < 0.01	*R*_s_ = 0.635 *p* < 0.01
	BPH	*R*_s_ = 0.008 *p* = 0.96	*R*_s_ = 0.802 *p* < 0.01	*R*_s_ = 0.587 *p* < 0.01	*R*_s_ = 0.435 *p* < 0.01
Omentin	PCa		*R*_s_ = 0.031 *p* = 0.85	*R*_s_ = −0.136 *p* = 0.40	*R*_s_ = −0.536 *p* = 0.74
	BPH		*R*_s_ = −0.039 *p* = 0.81	*R*_s_ = −0.006 *p* = 0.99	*R*_s_ = −0.019 *p* = 0.91
VEGF	PCa	*R*_s_ = 0.0310 *p* = 0.85		*R*_s_ = 0.848 *p* < 0.01	*R*_s_ = 0.867 *p* < 0.01
	BPH	*R*_s_ = −0.039 *p* = 0.81		*R*_s_ = 0.775 *p* < 0.01	*R*_s_ = 0.561 *p* < 0.01
HGF	PCa	*R*_s_ = −0.136 *p* = 0.40	*R*_s_ = 0.848 *p* < 0.01		*R*_s_ = 0.755 *p* < 0.01
	BPH	*R*_s_ = −0.006 *p* = 0.96	*R*_s_ = 0.775 *p* < 0.01		*R*_s_ = 0.626 *P* < 0.01
PSA	PCa	*R*_s_ = 0.049 *p* = 0.66	*R*_s_ = 0.867 *p* < 0.01	*R*_s_ = 0.755 *p* < 0.01	
	BPH	*R*_s_ = −0.019 *p* = 0.91	*R*_s_ = 0.561 *p* < 0.01	*R*_s_ = 0.626 *p* < 0.01	

*R*_S_ = Spearman's rank correlation coefficient, *p* = significance level.

**Table 4:** Multiple stepwise logistic analysis showing factors independently associated with PCa.

Final step—independent variables: PSA, omentin					
Significance level: *p* < 0.01					
Variable	Coefficient	Standard error	*p*	Odds ratio	95% confidence interval
PSA	0.088	0.027	<0.01	1.091 (59.8)	1.036 to 1.150
Omentin	0.005	0.002	0.01	1.005 (56.9)	1.001 to 1.009
Constant	−3.611	1.061	<0.01		

## Data Availability

The anonymized medical histories and epicrises of patients used to support the findings of this study may be obtained upon application to the Public Health Care Provincial Specialist Hospital No. 3 in Rybnik, who can be contacted at St. Energetyków 46, 44-200, Rybnik, Poland, Tel/Fax; +48 32 42 28 272.
